# Synthetic Polyclonal-Derived CDR Peptides as an Innovative Strategy in Glaucoma Therapy

**DOI:** 10.3390/jcm8081222

**Published:** 2019-08-15

**Authors:** Carsten Schmelter, Kristian Nzogang Fomo, Natarajan Perumal, Caroline Manicam, Katharina Bell, Norbert Pfeiffer, Franz H. Grus

**Affiliations:** Department of Experimental and Translational Ophthalmology, University Medical Center, Johannes Gutenberg University, 55131 Mainz, Germany

**Keywords:** glaucoma, autoimmunity, synthetic CDR peptides, neuroprotection, HTRA2, *Sus scrofa domestica*

## Abstract

The pathogenesis of glaucoma is strongly associated with the occurrence of autoimmune-mediated loss of retinal ganglion cells (RGCs) and additionally, recent evidence shows that specific antibody-derived signature peptides are significantly differentially expressed in sera of primary-open angle glaucoma patients (POAG) compared to healthy controls. Synthetically antibody-derived peptides can modulate various effector functions of the immune system and act as antimicrobial or antiviral molecules. In an ex vivo adolescent glaucoma model, this study, for the first time, demonstrates that polyclonal-derived complementarity-determining regions (CDRs) can significantly increase the survival rate of RGCs (*p* = 0.013). We subsequently performed affinity capture experiments that verified the mitochondrial serine protease HTRA2 (gene name: *HTRA2*) as a high-affinity retinal epitope target of CDR1 sequence motif *ASGYTFTNYGLSWVR.* Quantitative proteomic analysis of the CDR-treated retinal explants revealed increased expression of various anti-apoptotic and anti-oxidative proteins (e.g., VDAC2 and TXN) compared to untreated controls (*p* < 0.05) as well as decreased expression levels of cellular stress response markers (e.g., HSPE1 and HSP90AA1). Mitochondrial dysfunction, the protein ubiquitination pathway and oxidative phosphorylation were annotated as the most significantly affected signaling pathways and possibly can be traced back to the CDR-induced inhibition or modulation of the master regulator HTRA2. These findings emphasize the great potential of synthetic polyclonal-derived CDR peptides as therapeutic agents in future glaucoma therapy and provide an excellent basis for affinity-based biomarker discovery purposes.

## 1. Introduction

Glaucoma is a neurodegenerative ocular disease characterized by the progressive loss of retinal ganglion cells (RGCs) and their axons, resulting in optic nerve damage and visual field defects [1]. Elevated intraocular pressure (IOP) is one of the most common risk factors for the development of glaucoma and can be detected in approximately 70% of all patients; it has been termed the primary open-angle glaucoma (POAG) [1]. Next to other factors, the participation of an autoimmune component, including autoantibodies (AAB), has become a focus of attention in glaucoma research, providing new attractive targets for future diagnostic or therapeutic purposes [2,3,4,5,6,7]. A multitude of AABs against different kinds of retinal or optic nerve antigens have been identified, such as several heat shock proteins (HSP27, HSP60 and HSP70) [8,9], various crystallins (α- and β-crystallin) [8,10], vimentin [10], glycosaminoglycans [11] and α-fodrin [12]. Interestingly, many of these auto-reactivities show a high degree of congruence in sera as well as aqueous humor of glaucoma patients [13,14], and also remain remarkably stable between different study populations [12]. Besides the great potential of these immune-related biomarker candidates for diagnostic applications [14], we have also provided important evidence for the neuroprotective effects of various AAB molecules (e.g., against GFAP, 14-3-3, α-and γ-synuclein) on RGCs in in vivo and ex vivo glaucoma models [15,16,17,18,19]. These findings underline the important therapeutic potential of AABs found in low abundant titers in glaucoma patients and could also serve as an additional treatment option in glaucoma therapy in combination with IOP-lowering medications. However, often, therapeutic macromolecules, including antibodies, have several disadvantages, such as immunogenic properties, poor tissue penetration and high manufacturing costs, thus limiting their application spectrum in daily clinical routine [20].

Liquid-chromatography mass spectrometry (LC-MS) represents a powerful instrument for reproducible identification and quantification of peptides from the variable domain of highly diverse antibodies without any prior knowledge about the targeted (auto-) antigens [21,22,23,24]. By implementation of this MS-based analytical approach we identified several polyclonal IgG V domain peptides, particularly complementarity-determining regions (CDR), which were significantly differentially expressed in the protein backbone of sera-derived IgG from POAG patients in comparison to healthy controls [25]. This procedure facilitates the direct sequencing of Ig-derived peptide motifs which are shared between several B cell clones. To its advantage, it is independent of predefined protein panels, which in contrary, are needed as the basis for AAB profiling via microarray or Western Blot analysis. However, CDRs are hypervariable sequence motifs (paratopes) of the variable Ig domain, and determine the active bindings sites of the antibodies which are primarily responsible for the antigen specificity [26]. Many studies have already highlighted the versatile biological functions of synthetic CDR-derived peptides, ranging from immunomodulatory or immunoregulatory effects [27,28]; moreover, to antiviral, antibacterial or antitumor activities [29,30,31], which are independent of the antigen specificity of the native antibodies. On the one hand, Rabaça et al., (2016) [32] demonstrated that a synthetic CDR-related peptide, encoding the VH CDR3 of murine monoclonal antibody AC 1001, displayed cytotoxic effects on murine and human melanoma cells ex vivo by inducing reactive oxygen species (ROS) and apoptotic signaling pathways. On the other hand, another synthetic tolerogenic CDR peptide showed ameliorating effects in different animal models for systemic lupus erythematosus (SLE) by triggering immunomodulatory and immunosuppressive activities [33,34,35], favoring the general application of synthetic CDR peptides as therapeutic agents in other autoimmune-related neurodegenerative diseases.

Based on these previous observations, the main objective of the present study was to evaluate if selected synthetic glaucoma-associated CDR1 peptides (*ASGYTFTNYGLSWVR* and *ASQSVSSYLAWYQQK*) may trigger any neuroprotective or even neuro-damaging events on RGCs in an ex vivo glaucoma model. The underlined part of the peptide indicates the CDR1 sequence motif. Furthermore, both CDR1 sequence motifs were screened for potential interaction partners (epitope targets) in the retinal porcine proteome by using state-of-the-art affinity capture LC-MS technologies. The presented study therefore provides important information about the applicability and effectiveness of synthetic CDR peptides in future glaucoma therapy, and aims to unravel the complex biological function of these highly specific immune-related biomarker candidates.

## 2. Experimental Section

### 2.1. Retina Isolation and Homogenization

Retina tissues were prepared from freshly removed eye bulbs (*n* = 20) from the house swine (*Sus scrofa domestica* Linnaeus, 1758; sacrificed at 3 to 6 months; female: male = 3:2) provided by local slaughterhouses (Landmetzgerei Harth, Stadecken–Elsheim, Germany; Hofgut Acker, Bodenheim, Germany). The use of animal by-products of the house swine for scientific research purposes was approved by the Kreisverwaltung Mainz-Bingen in Germany (Identification Code: DE 07 315 0006 21, approved on 13 January 2014). Preparation of the eye bulbs was performed under sterile conditions no later than 3 h after slaughtering. First, the eye bulbs were disinfected with 70% ethanol (EtOH) and subsequently radially opened with a scalpel to remove lens, vitreous body, iris and ciliary body. Next, retina tissues were carefully separated from the retinal pigment epithelium (RPE) with a paintbrush and separated from the optic nerve head with fine scissors. Samples were transferred into 2 mL screw cap microtubes, snap-frozen in liquid nitrogen and stored at −80 °C. Prior to homogenization, 1.4/2.8 mm ceramic balls (VWR International GmbH, Darmstadt, Germany) were added to frozen retinal tissues and filled with 1 mL Tissue Protein Extraction Reagent (T-PER, Thermo Fisher Scientific, Rockford, IL, USA). Retina samples were subjected for homogenization with the Precellys^®^ 24 homogenizer (VWR International GmbH, Darmstadt, Germany) for 45 s three times, at 5000 rpm. Retinal homogenates were centrifuged at 10,000 *g* for 12 min at 4 °C and the supernatant was collected into new 2 mL reaction tubes. To avoid sample contamination by insoluble cell components the centrifugation step was repeated once again, and the supernatant containing soluble retinal proteins was exchanged into 300 µL phosphate-buffered saline (PBS) using an Amicon 3 kDa centrifugal filter device (Millipore, Billerica, MA, USA). The concentrated protein lysates were pooled and protein measurements were performed using a Pierce BCA Protein Assay Kit (Thermo Fisher Scientific, Rockford, IL, USA) according to the manufacturer’s protocol. The protein lysate pool was diluted in the ratios of 1:20, 1:30 and 1:40 in PBS (*v*/*v*) and measured three times using a Multiscan Ascent photometer (Thermo Fisher Scientific, Rockford, IL, USA) at a wavelength of 570 nm. The protein lysate pool was divided into 5 mg aliquots (in 200 µL PBS) and stored at −80 °C.

### 2.2. CDRs and a Scrambled Peptide as Control Peptides

Two complementarity-determining regions (CDRs), which were associated with glaucoma [25], were synthesized in cooperation with the Translational Oncology Mainz of the Johannes Gutenberg University (TRON, Mainz, Germany) and PEPSCAN (Lelystad, Netherlands). CDR peptide sequences were synthesized as follows: *ASGYTFTNYGLSWVR* (CDR1) and *ASQSVSSYLAWYQQK* (CDR1). At first, both CDR peptides were synthesized with an N-terminal Biotin-[TTDS] linker for epitope identification experiments (see Section 2.3). CDR peptides (*ASGYTFTNYGLSWVR*) with successfully identified interaction partners were further synthesized without modification along with their respective scrambled peptide analogs as controls (*YVWAGSTLSRTGNFY*; without modification and with an N-terminal Biotin-[TTDS] linker)

### 2.3. Identification of CDR-Specific Epitope Targets

The synthetic CDR peptides with N-terminal Biotin-[TTDS] linkers were immobilized on Pierce™ Streptavidin Magnetic Beads (Thermo Fisher Scientific, Rockford, IL, USA) according to the supplier’s protocol (*n* = 3). In brief, 50 µL of the magnetic beads were washed two times with 200 µL PBS using a magnetic stand and labeled for 1 h at room temperature (RT) with 80 µg of the N-terminal biotin-labeled synthetic peptides. As a control group (*n* = 3), biotin-labeled (0.5 mg/mL) magnetic beads were included in the experiment to distinguish non-specific from CDR-specific binders. After peptide immobilization, magnetic bead fractions (*n* = 3) were washed twice with PBS followed by incubation and gentle mixing with 5 mg homogenized pig retina (see Section 2.1) at 4 °C overnight. On the next day, the unbound protein fraction was discarded and the labeled magnetic beads were extensively washed with 300 µL PBS three times to diminish non-specific bindings. The remaining attached proteins were eluted with 100 µL Pierce^TM^ IgG Elution Buffer pH 2.0 (Thermo Fisher Scientific, Rockford, IL, USA) and transferred into reaction tubes containing 10 µL 1M Tris HCl pH 8.5. The eluate fractions were evaporated in the SpeedVac (Eppendorf, Darmstadt, Germany) for 30 min at 30 °C until dryness and stored at −20 °C prior to further in-solution trypsin digestion. A spike-in experiment for the verification of the interaction partner, recombinant HTRA2/Omi (Human Serine Protease, cat. no. C760) was purchased from Novoprotein (Summit, NJ, USA). The spike-in experiment also included a scrambled peptide analog as proper control to confirm sequence specificity of the CDR peptide-protein interaction.

### 2.4. Retinal Explants and Immunohistology

The adolescent retina organ culture (*Sus scrofa domestica* Linnaeus, 1758) represents an excellent ex vivo glaucoma model and was already used to study the neuroprotective effects of specific AABs on RGCs [17]. The optic nerve cut (ONC) during slaughtering initiated the neurodegeneration process and led to significant RGC loss within 24 h of incubation. Preparation of the retina-RPE complexes (5 × 5 mm) was performed in accordance with previous publication [17] and were cut in the dorsal periphery above the visual streak to ensure homogeneous distribution of RGCs in each experiment. Afterwards, the retinal explants were cultured in neurobasal A medium supplemented with 2% B27, 1% N2, 0.8 mM L-alanyl-L-glutamine and 1% penicillin/streptomycin in an incubator at 37 °C and 5% CO2 for 24 h. Control explants were cultured for 24 h without any synthetic peptides (*n* = 4). In addition, further retinal explants were treated either with 25 µg/mL synthetic CDR peptide (*ASGYTFTNYGLSWVR*, *n* = 4) or 25 µg/mL scrambled peptide analog (*YVWAGSTLSRTGNFY*, *n* = 4) as described in previous publications [36,37]. After cultivation, control and treated retinal sections were carefully removed from the RPE, washed twice with PBS and fixed in 4% paraformaldehyde (PFA) for 30 min. Retinal flatmounts were again washed twice with PBS and subsequently blocked and permeabilized for 2 h with 200 µL 0.3% Triton-X-100 (Sigma-Aldrich, St. Louis, MO, USA) and 10% fetal calf serum (Merck Millipore, Darmstadt, Germany) in PBS. After discarding the blocking buffer, the flatmounts were stained with 1:250 goat anti-Brn3a (Santa Cruz Biotechnology, Dallas, TX, USA) overnight at 4 °C. Next day, the retinal flatmounts were washed twice with PBS and incubated with 1:400 secondary Alexa Flour 568 donkey anti-goat (H+L; Thermo Fisher Scientific, Rockford, IL, USA) for 2 h in the dark. Subsequently, after the staining, a TUNEL assay using the In Situ Cell Death Detection Kit, Fluorescein (Roche, Basel, Switzerland) was performed according to the supplier’s protocol at 37 °C for 1 h. Retinal flatmounts were washed three times with PBS and stained with 1:2500 4′,6-Diamidin-2-phenylindol (DAPI; Thermo Fisher Scientific, Rockford, IL, USA) in PBS for 5 min at RT. After washing, the retinal flatmounts were mounted with vector shield mounting medium (Vector Laboratories, Burlingame, CA, USA) on Superfrost Plus™ slides (Thermo Fisher Scientific, Rockford, IL, USA). Fluorescence microscopy was performed with a Nikon Eclipse TS100 microscope (Nikon Instruments, Tokyo, Japan) combined with a DS-Fi1-U2 digital camera and NIS elements software. Eleven high-resolution pictures (20-fold magnification) were taken from each the retinal flatmount at different positions, resulting in 44 pictures per experimental group (TRITC, FITC and DAPI channel). High-resolution fluorescent images were analyzed randomized via open-source ImageJ software package [38] by experienced laboratory personnel. Brightness and contrast of the high-resolution fluorescent images were adjusted to facilitate the manual counting of the Brn3a^+^ and TUNEL^+^ cells. The number of RGCs (Brn3a^+^ cells) was extrapolated to RGC/mm^2^ and the percentage distribution of TUNEL^+^ RGC was calculated.

### 2.5. In-Gel and In-Solution Trypsin Digestion

For the identification of the CDR-specific interaction partners, the eluate fractions (see Section 2.3) were subjected to further in-solution trypsin digestion as described elsewhere [25,39,40]. In brief, the eluate fractions were dissolved in 30 µL of 50 mM ammonium bicarbonate (ABC) and sonicated for 5 min on ice. Subsequently, 6 µL of 100 mM dithiothreitol (DTT) in 50 mM ABC were added and incubated for 30 min at 56 °C. Next, 6 µL of 200mM iodoacetamide (IAA) in 50 mM ABC were added and incubated for 30 min at RT in the dark. The reduced and alkylated proteins were digested overnight with 0.2 mg/mL trypsin (Promega; Madison, WI, USA) in 50 mM ABC 10% acetonitrile (ACN) at 37 °C. The next day, the digestion was quenched with 10 µL of 0.1% formic acid (FA), evaporated in the SpeedVac for 30 min at 30 °C until dryness and stored at −20 °C. To unravel the CDR-induced proteomic changes in the retinal explants (see Section 2.4), in-gel trypsin digestion for mass spectrometry (MS)-based proteomics was performed. CDR-treated and untreated retinal explants (*n* = 3 per group) were transferred into 2 mL screw cap microtubes and subsequently frozen in liquid nitrogen. Frozen explants were filled with 400 µL T-PER buffer and 1.4/2.8 mm ceramic balls were added. Protein extraction was performed using the Precellys^®^ 24 homogenizer and homogenates were exchanged in 200 µL PBS by using an Amicon 3 kDa centrifugal filter device (as described in detail in Section 2.1). Total protein amounts of the retinal explants were determined by a Pierce BCA Protein Assay Kit and Multiscan Ascent photometer. Up to 40 µg of protein lysate per explant were separated under reduced conditions on 10-well NuPAGE 12% Bis-Tris minigels (Thermo Fisher Scientific, Rockford, IL, USA) by using NuPAGE™ MOPS SDS Running Buffer 20X (Thermo Fisher Scientific, Rockford, IL, USA) in accordance with the supplier’s protocol. In case of the verification experiment (spike-in experiment), the whole eluate fractions of the different bead groups (CTRL beads, beads labeled with scrambled peptide and CDR peptide; *n* = 3) were separated by 1-D SDS PAGE. Gels were run at 150 V for 1.5 h at 4 °C and subsequently stained by using the Novex Colloidal Blue Staining Kit (Thermo Fisher Scientific, Rockford, IL, USA) according to the manufacturer’s instructions. Gels were de-stained overnight and scanned using an Epson Perfection V600 Photo Scanner (Seiko Epson Corporation; Suma, Nagano, Japan) at 700 dpi. Each lane of the CDR-treated and untreated retinal explants was subdivided into 17 slices and subjected to further in-gel trypsin digestion as described in previous publications [39,41,42,43]. In case of the verification experiment, only the synthetic HTRA2-contraining proteins spots (above ≈49 kDa) were subjected to further in-gel trypsin digestion. Prior to LC-MS/MS analysis the tryptic peptides of the in-solution and in-gel digest were purified by SOLAµ™ HRP SPE spin plates (Thermo Fisher Scientific, Rockford, IL, USA) according to the manufacturer’s protocol [44].

### 2.6. LC-MS/MS Analysis

LC-MS/MS measurements were performed with the Hybrid Linear Ion Trap-Orbitrap MS system (LTQ Orbitrap XL; Thermo Fisher Scientific, Rockford, IL, USA). as described in detail elsewhere [25,39,41,43,44]. Solvent A consisted of 0.1% formic acid (FA) in water and solvent B consisted of 0.1% FA in ACN. Peptides of the in-solution tryptic digest were eluted within 120 min using following gradient program: 10–20% B (0–5 min), 15–20% B (5–15 min), 20–35% B (15–85 min), 35–90% B (85–105 min) and 10% B (105–120 min). On the contrary, peptides of the in-gel tryptic digest were eluted within 60 min using following gradient program: 15–40% B (0–30 min), 40–60% B (30–35 min), 60–90% B (35–45 min) and 10% B (45–60 min). The LTQ Orbitrap operated with a resolution of 30,000 in the positive ion mode and the target automatic gain control (AGC) was set to 1 × 10^6^ ions. The lock mass for internal calibration was set to 445,120,025 m/z (polydimethylcyclosiloxane). Dynamic exclusion (DE) mode was enabled with the following settings for the in-solution digest: Repeat count = 2, repeat duration = 30 s, exclusion list size = 250, exclusion duration = 300 s and exclusion mass width of ±10 ppm. For the in-gel digest the DE setting exclusion duration was set to 90 s. The high-resolution MS scan of the Orbitrap-FTMS analyzer provided the selection of the 5 most intense peptide ions for collision induced dissociation (CID) fragmentation in the ion trap employing a normalized collision energy of 35%. Quality control of the total ion current (TIC) chromatogram was performed by using Qual Browser v. 2.0.7 SP1 (Thermo Fisher Scientific, Rockford, IL, USA). The mass spectrometry proteomics data have been deposited to the ProteomeXchange Consortium via the PRIDE [45] partner repository with the dataset identifier PXD014718.

### 2.7. Protein Identification and Quantification

For protein identification and quantification, the acquired tandem MS spectra were analyzed with the computational proteomics platform MaxQuant version 1.6.1.0 (Max Planck Institute of Biochemistry, Martinsried, Germany). Output data were searched against SwissProt databases with the taxonomies *Homo sapiens* (Date: 07/18/2018, 20.385 sequences) and *Sus scrofa* (Date: 18 July 2018, 1424 sequences) with the following settings: Peptide mass tolerance of ±30 ppm, fragment mass tolerance of ±0.5 Da, tryptic cleavage, a maximum of two missed cleavages, carbamidomethylation as fixed modification, and acetylation (Protein N-terminal) and oxidation as variable modifications of methionine. In addition, proteins were filtered with a false discovery rate (FDR) of <1% and the MaxQuant specific feature “match between run” was enabled.

### 2.8. Data Analysis and Bioinformatics

Statistical analysis of the MaxQuant generated output data (“proteins.txt”) was performed by using the software program Perseus, version 1.5.5.0 (Max Planck Institute of Biochemistry, Martinsried, Germany). At first, intensities of the detected proteins were log2 transformed prior to further analysis. Data matrix was filtered for contaminants, reversed hits and “only identified by site”. Proteins had to be detected in at least three replicates of one study group, and identification based on two peptides was accepted. Missing intensity values were imputed by random numbers received from the normal distribution (width: 0.3, down shift: 1.8). For the identification of CDR-specific interaction partners (see Section 2.3) only missing values of the control bead group were imputed in accordance with previous publication [46]. Afterwards, two-sided *t*-test statistics with *p*-values <0.05 were applied in order to identify significantly changed protein species. For the illustration of the heat map the log2 transformed protein abundances were standardized by z-score. The heat map was generated with hierarchical clustering based on Euclidean distance using the Perseus software package. Further statistical analyses and graphical presentation of the data were performed by using Statistica version 13 (Statsoft; Tulsa, OK, USA) or EXCEL 2013 functions. Functional annotation and pathway analyses were performed with web-based biological database STRING version 10.5 (Search Tool for the Retrieval of Interacting Genes/Proteins) and Ingenuity Pathway Analysis software version 1-04 (IPA, Ingenuity QIAGEN; Redwood City, CA, USA), as described in detail in previous publications [40,47].

## 3. Results

### 3.1. Identification of CDR-Specific Epitope Targets

**Figure 1:**
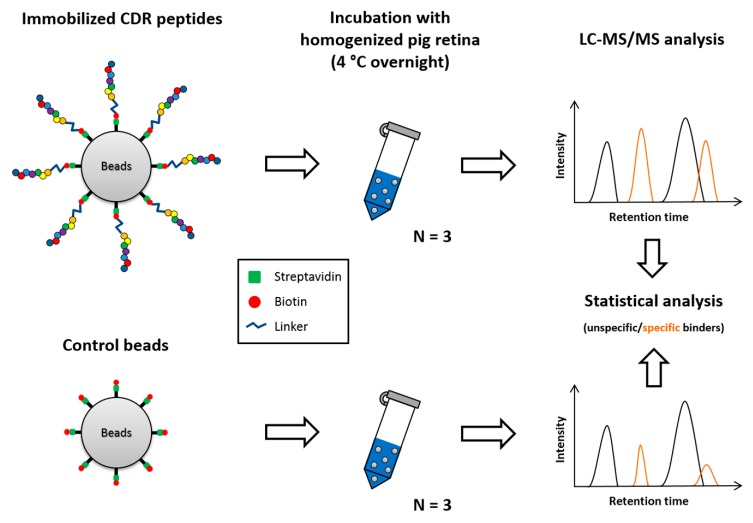
Schematic illustration showing affinity capture experiments of potential epitope targets by synthetic complementarity-determining regions (CDR)-derived peptides. CDR peptides were synthesized with an N-terminal [TTDS]-biotin modification by a specialist manufacturer. For epitope identification the modified synthetic peptides (80 µg) were attached to commercially available streptavidin beads and incubated with 5 mg homogenized pig retina. To distinguish non-specific from specific protein binders a biotin-labeled control group was included in the analysis. After incubation, all bead fractions were extensively washed and the remaining attached proteins were eluted by pH shift. Eluate fractions were subjected to further in-solution trypsin digestion and analyzed by LC-MS/MS. Statistical analysis revealed high-affinity epitope targets (interaction partners) of the synthetic CDR peptides.

To prove the concept and the feasibility of the MS-based epitope identification workflow (see Figure 1 and methods Section 2.3), we selected two CDR1 peptide sequences found as low abundant in glaucoma patients for the affinity capture experiment. Both CDR1 sequence motifs (*ASGYTFTNYGLSWVR* homologous to IGHV1-18*02 and *ASQSVSSYLAWYQQK* homologous to IGKV3-11*01) were significantly lower expressed in the protein backbone of polyclonal, sera-derived IgG molecules of glaucoma patients in contrast to healthy controls and provided attractive targets for the MS-based epitope identification [25]. Binding efficiencies of both synthetic N-terminal biotinylated CDR1 peptides to magnetic streptavidin beads were confirmed by LC-MS (see Figure S1).

**Figure 2:**
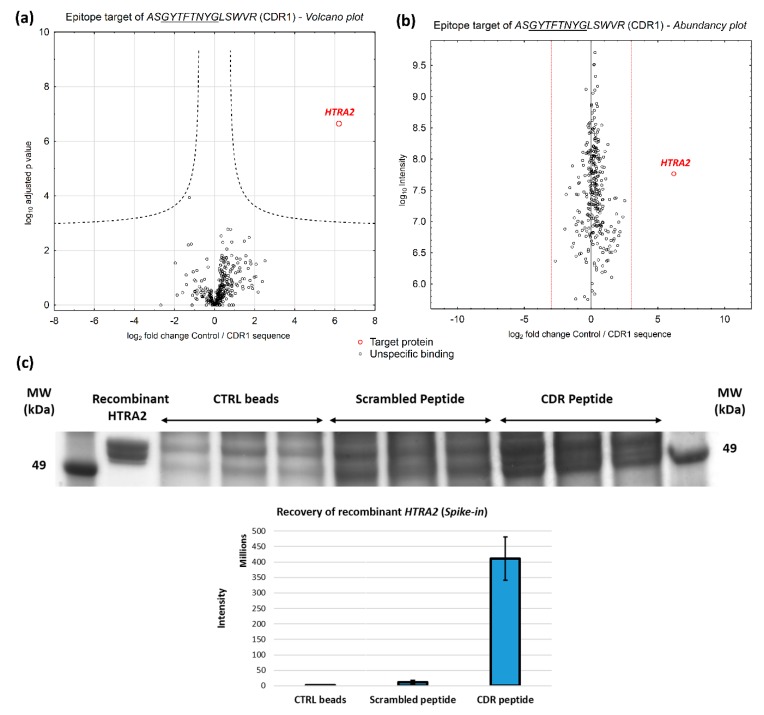
Identification of potential epitope targets of the CDR peptides by affinity-based proteomic strategy with 5 mg homogenized pig retina. (**a**) Volcano plot showing log2 fold change plotted against –log10 adjusted *p* values for samples from CDR-labeled bead group (*n* = 3), versus samples from control bead group (*n* = 3) (*p* < 0.001; log2 fold change >3). Mitochondrial serine protease HTRA2 was identified as high-affinity interaction partner for the synthetic CDR1 peptide *ASGYTFTNYGLSWVR*. (**b**) Abundancy plot showing log2 fold change plotted against –log10 adjusted cumulative intensity. HTRA2 represents a rare protein in the porcine retina and confirms the specific interaction with the synthetic CDR1 peptide. (**c**) Spike-in experiment of 2 µg recombinant HTRA2 in 5 mg homogenized pig retina followed by affinity-based proteomics. The image of the 1-D SDS PAGE shows the eluate fractions of the different bead groups (Control (CTRL) beads and beads labeled either with scrambled peptide or CDR peptide, *n* = 3). In-gel trypsin digestion and LC-MS/MS revealed that CTRL beads and the scrambled peptide analog recovered far lesser quantities of recombinant HTRA2 in contrast to the original CDR peptide and confirms the sequence specificity of the interaction with HTRA2.

Affinity capture experiments revealed that mitochondrial serine protease HTRA2 (gene name: *HTRA2*) represents a high-affinity interaction partner for the CDR1 sequence motif *ASGYTFTNYGLSWVR* (see Figure 2a and File S1, *p* < 0.001 and log2 fold change >3). In addition, epitope target HTRA2 shows an overall low protein abundance (cumulative intensity) in the entire retinal porcine proteome. (Figure 2b) and confirms the specific interaction behavior with the glaucoma-associated CDR1 peptide. For further verification of the specific CDR1 peptide-protein interaction, we repeated the affinity capture experiments with an additional spike-in of 2 µg recombinant HTRA2 into the homogenized pig retina. In addition, a scrambled peptide analog was included in this experiment to prove the sequence specificity of the CDR 1 peptide-protein interaction. To maximize the quantitative protein recovery, we performed an in-gel trypsin digestion of the synthetic HTRA2-containing protein spots (above 49 kDa) followed by LC-MS/MS analysis (see method Section 2.5 and Figure 2c). For the final result, the control beads (intensity: 2.9 × 10^6^ ± 1.8 × 10^6^), as well as the scrambled peptide analog (intensity: 1.4 × 10^7^ ± 6.3 × 10^7^) enriched much lower amounts of recombinant HTRA2 compared to the original CDR peptide (intensity: 4.2 × 10^8^ ± 7.1 × 10^7^) which highlights the importance of the unique sequence specificity for the CDR1 peptide-protein interaction (see Figure 2c). However, no significant interaction partner for the CDR1 sequence motif *ASQSVSSYLAWYQQK* was identified by affinity capture experiments (*p* < 0.001 and log2 fold change >3), possibly due to insufficient binding efficiency to the magnetic beads (see Figures S1 and S2). For that reason, only CDR1 peptide sequence *ASGYTFTNYGLSWVR* was used for further experimental analyses.

### 3.2. CDR-Induced Effects in an Ex Vivo Glaucoma Model

**Figure 3:**
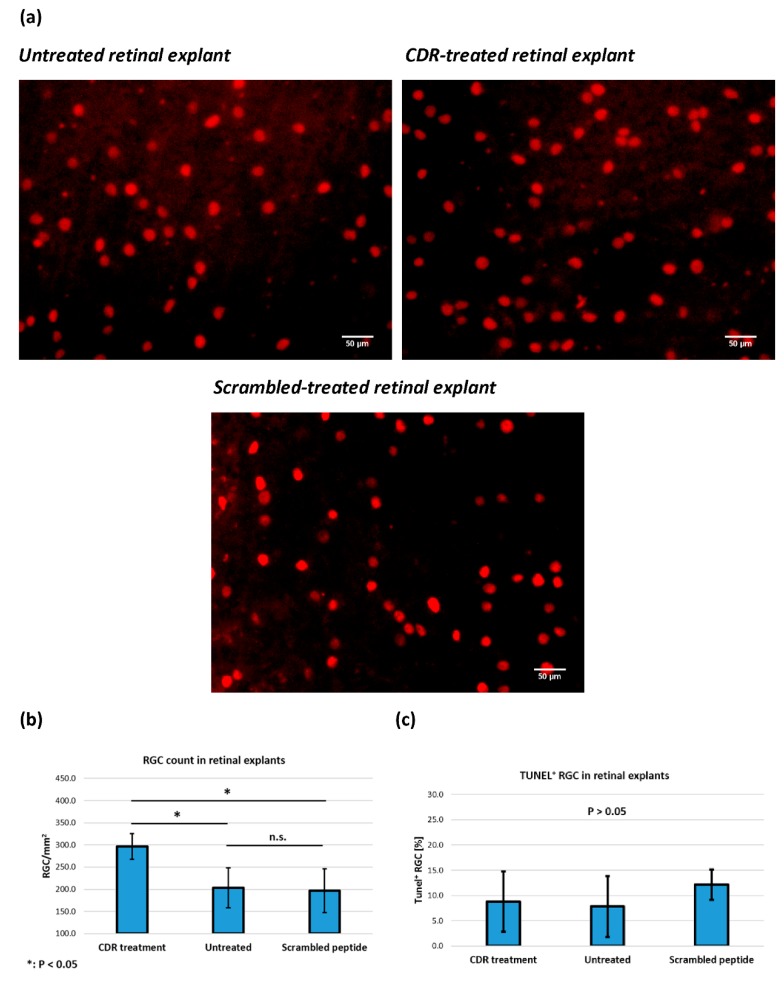
Effect of the CDR peptide on the number of retinal ganglion cells (RGCs) revealed by Brn3a^+^ staining and the percentage of apoptotic RGCs determined by TUNEL assay. Retinal explants (*n* = 4 per group) were cultivated with control medium without any peptide (untreated control) or with medium with additional 25 µg/mL CDR peptide for 24 h. Furthermore, further retinal explants were also incubated in medium with 25 µg/mL scrambled peptide analog serving as proper control. (**a**) Brn3a^+^ staining of retinal flatmounts without any treatment, treated with the CDR peptide or treated with the scrambled peptide analog. (**b**) CDR-treated explants showed significant higher RGC survival in contrast to the untreated control group and scrambled peptide group (*p* < 0.05). (**c**) Quantitative analysis of TUNEL^+^ RGC did not show any significant difference between the groups (*p* > 0.05).

To address the question of whether the synthetic CDR1 sequence motif *ASGYTFTNYGLSWVR* may trigger any neuroprotective or even neuro-damaging activities in glaucoma, we used an established ex vivo glaucoma model for evaluation. The adolescent porcine retina organ culture [17] provides excellent requirements to study CDR-induced effects on RGCs after an optic nerve cut (ONC) and is suitable to assess the effectiveness of synthetic CDR peptides as potential drug candidates in future glaucoma therapy. The immunohistochemical Brn3a staining represents an established method in our laboratory and is routinely used for the evaluation of neuroprotective effects on RGCs during stress conditions [19,48,49,50]. And indeed, quantitative analysis of RGC/mm^2^ (also Brn3a^+^ cells/mm^2^, see Figure 3a,b) revealed a significantly higher RGC survival rate in CDR-treated retinal explants (296 ± 29 RGC/mm^2^, *n* = 4) in contrast to untreated control explants (203 ± 45 RGC/mm^2^, *n* = 4, *p* = 0.013).

In addition, a scramble peptide analog (*YVWAGSTLSRTGNFY*) was used as a proper control in further retinal explants (197 ± 50 RGC/mm^2^, *n* = 4, *p* = 0.014) and verified the sequence specificity of the CDR-induced, neuroprotective effects on RGCs. However, no significant differences in the percentage distribution of TUNEL^+^ RGCs (see Figure 3c) were found between all three experimental groups (Untreated control: 8.8 ± 6.0%, CDR: 7.9 ± 6.0% and scrambled peptide: 12.2 ± 3.0%, *p* > 0.05).

### 3.3. CDR-Induced Proteomic Changes in Retinal Explants

**Figure 4:**
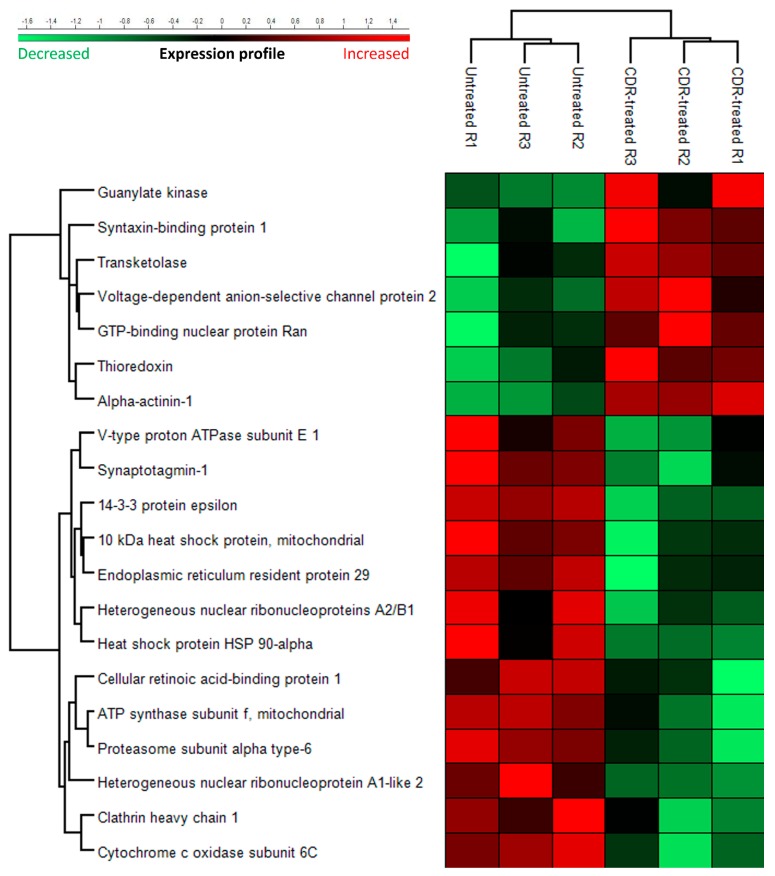
Heat map showing the most significant proteomic changes (*p* < 0.05) in retinal explants (*n* = 3 per group) cultivated with medium without any peptide (untreated control) or with medium with 25 µg/mL CDR peptide for 24 h after the optic nerve cut (ONC).

**Figure 5:**
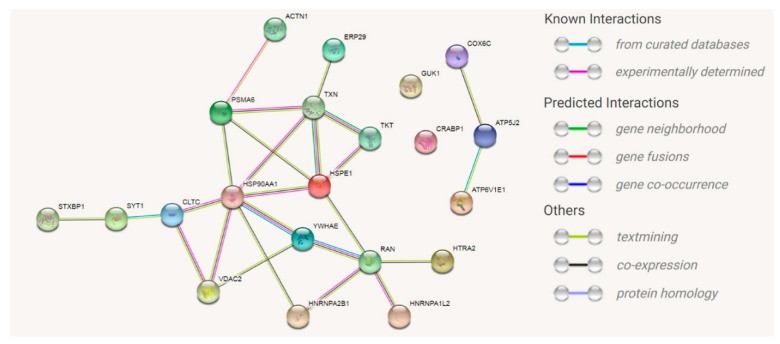
Analysis of the CDR-induced signaling pathways in the retinal explants after 24h of incubation. Search Tool for the Retrieval of Interacting Genes/Proteins (STRING) shows the signaling pathways of the most significantly changed proteins using the medium confidence score (0.4). Epitope target HTRA2 shows at least a text-mining and co-expression to GTP-binding nuclear protein RAN.

To unravel the neuroprotective effects of the CDR1 sequence motif *ASGYTFTNYGLSWVR* on RGCs, we performed LC-MS based quantitative proteomics of the untreated and CDR-treated retinal explants (*n* = 3 per group; see File S2). In total 354 retinal proteins were identified in both experimental groups with an FDR <1%. Up to 6% of all identified proteins showed a significant change in expression level (*p* < 0.05) between the CDR-treated retinas and the untreated control explants (see Figure 4). Interestingly, representative stress response protein markers, such as heat shock protein HSP-90 alpha (gene name: *HSP90AA1*), 10kDa heat shock protein mitochondrial (gene name: *HSPE1*), endoplasmic reticulum resident protein 29 (gene name: *ERP29*) or cytochrome c oxidase subunit 6C (gene name: *COX6C*) were significantly lower expressed in CDR-treated explants in contrast to untreated controls. On the other hand, proteins with neuroprotective or anti-oxidative properties such as voltage-dependent anion-selective protein channel 2 (gene name: *VDAC2*), GTP-binding nuclear protein Ran (gene name: *RAN*) or thioredoxin (gene name: *TXN*) were significantly more abundant in CDR-treated samples in comparison to untreated controls. Additionally, other attractive protein markers, such as carbonic anhydrase 2 (gene name: *CA2*), putative elongation factor 1-alpha-like 3 (gene name: *EEF1A1P5*) or endoplasmin (gene name: *HSP90B1*) showed at least a tendency to being differentially expressed between both study groups (*p* < 0.1; see Figure S3). However, CDR1-specific epitope target HTRA2 (see Figure 2) was not detected by LC-MS/MS, probably because of the limits of detection. Nevertheless, functional annotation and pathway analysis confirmed a hypothetical interaction or regulation of the CDR-induced signaling pathways by HTRA2 (see Figure 5).

IPA analysis revealed that the top five most significant canonical pathways include mitochondrial dysfunction, protein ubiquitination pathway, oxidative phosphorylation, PI3K/AKT signaling and the thioredoxin pathway (see Table 1). Furthermore, most of the significantly differentially expressed proteins participate in various biological activities; namely, post-translational modifications, protein folding, molecular transport, protein trafficking and maintenance of cellular functions (see Table 2 and File S3).

## 4. Discussion

Immunopeptidomics is one of the fastest growing research areas with considerable progress in the field of personalized cancer immunotherapy [51]. As the term “immunopeptidomics” is generally associated with the MS-based identification of tumor-specific neoantigens serving as the basis for the targeted cancer therapy [51], the present study offers completely new treatment strategies for various autoimmune-related diseases, with a special focus on glaucoma. Recently, short synthetic CDR peptides have been commonly used as remarkably active biomolecules that trigger a wide range of effector functions, such as immunomodulatory, antimicrobial, antiviral or antitumor activities [27,28,29,30,31]. Synthetic CDR peptides have excellent properties as therapeutic agents. This is mainly due to their small size, low immunogenicity, good tissue penetration characteristics, and their ease and cost-effective manufacturing, with structural modification possibilities [52]. In comparison to the present work, the previous studies referred to CDR peptides of already sequenced antibody molecules with known biological activity or antigen specificity and never focused on polyclonal-derived CDR sequences with unknown biological function.

In the present study, two CDR1 sequence motifs (*ASGYTFTNYGLSWVR* and *ASQSVSSYLAWYQQK*), which we found in low abundance in the sera of glaucoma patients [25], were investigated for potential interaction partners (or epitope targets) in the retinal porcine proteome and screened for their potential neuroprotective effects on RGCs in an ex vivo glaucoma model. The established epitope identification workflow (see Figure 1) revealed the mitochondrial serine protease HTRA2 (gene name: *HTRA2*, see Figure 2) as a high-affinity interaction partner of the CDR1 sequence motif *ASGYTFTNYGLSWVR*. On the one hand, HTRA2 has drawn attention as an important, key player in apoptotic processes, promoting the degradation of anti-apoptotic proteins (e.g., X-linked inhibitor of apoptosis protein, gene name: *XIAP*) and the activation of caspase-dependent and independent pathways [53,54,55]. Thereby, during cellular stress responses, HTRA2 is increasingly released from the mitochondria to the cytosol [53,54,55] and has also been found in rat retina after optic nerve crushing [56]. On the other hand, recent studies also indicated that transgenic *htra2^mnd2^* mice deficient in HTRA2 activity, showed early signs of onset neurodegeneration, multiple tissue atrophy and early lethality, thereby, strengthening the importance of the role of HTRA2 in neuronal cell survival and mitochondrial homeostasis [57,58,59,60]. In accordance, dysfunction of HTRA2 protein activity is also associated with other neurodegenerative diseases, such as Parkinson’s or Alzheimer’s disease and represents an interesting target in cancer therapy [53,61]. Inhibition of HTRA2 activity, particularly the catalytic protein domain, prevented myocardial dysfunction and inflammation-mediated tissue damaging events after ischemia/reperfusion injury in rat hearts in vivo [62]. However, for glaucoma, the exact role of HTRA2 is still unknown and represents a very attractive target for future glaucoma-related study designs. Ding et al., (2009) [63] highlighted the importance of HTRA2-mediated apoptotic processes in murine RPE cells during oxidative stress conditions and proposed HTRA2 as one of the key players in the pathogenesis of the age-related macular degeneration (AMD). Due to that reason, it is not far-fetched to speculate that HTRA2 might also inherit important key functions in other eye-related disorders.

For the first time, we could demonstrate CDR-induced, neuroprotective activities of sequence motif *ASGYTFTNYGLSWVR* on RGCs ex vivo (see Figure 3a,b). The sequence specificity of these effects was verified by using a scrambled peptide analog as proper control. The number of RGC/mm^2^ (Brn3a^+^ cells/mm^2^) in the dorsal periphery of the porcine retina was in the range of previous publications [64,65] and confirms the reliability of the obtained results. However, no significant differences in the number of apoptotic cells were observed between all three experimental groups (*p* > 0.05, see Figure 3c). In general, apoptotic processes are largely dependent on the cell type, as well as environmental stimuli and can vary time-wise from 12 to 24 h [66,67]. Since the TUNEL assay only indicates the last stage of apoptosis by in situ detection of fragmented DNA and just covers a period about 2 to 3 h [68,69], the considered time frame could be too short to detect any significant changes in the cell death rate between the different groups. Nevertheless, considering the quantitative proteomic results (see Figure 4), we identified an increased expression of anti-apoptotic and anti-oxidative proteins (e.g., VDAC2 and TXN) in the CDR-treated retinal explants in comparison to the untreated controls accompanied by decreased levels of proteins associated with the cellular stress response (e.g., HSPE1, ERP29 and HSP90AA1). Mitochondrial VDAC2 plays an important role in cell homeostasis through ROS neutralization [70] and triggers anti-apoptotic functions by direct inhibition of pro-apoptotic BAK [71]. In accordance, the protein TXN is responsible for mitochondrial redox homeostasis and acts as important regulator of the energy metabolism in neuronal cells [72]. On the contrary, the proteins HSP90AA1, HSPE1 and ERP29 are mainly functional as molecular chaperone complexes essential for the maintenance of the protein folding and protein trafficking, (see Table 2 and File S3) during cellular stress responses and are a hallmark for various neurodegenerative diseases, including glaucoma [73,74,75]. Mitochondrial dysfunction, the protein ubiquitination pathway and oxidative phosphorylation (Table 1) were annotated as the three main affected canonical pathways and are recognized as the primarily responsible pathological factors in glaucoma [76,77,78,79]. As outlined above, HTRA2 plays an essential role in mitochondrial homeostasis by way of protein quality control (PQC) [57,58,59,60], and also directly inhibits E3 ubiquitin ligase activity [80,81], which strengthens our hypothesis that HTRA2 may act as master regulator of the three most affected canonical pathways. Whether the HTRA2 protein activity is mainly regulated by direct interaction (inhibition) with CDR1 sequence motif *ASGYTFTNYGLSWVR* remains to be determined. In addition, the oxidative and non-oxidative pentose phosphate pathways (PPP) also affected in the CDR-treated retinal explants (see Table 1) are mainly regulated by the increased expression of the protein transketolase (TKT) [82]. Kuehne et al., (2015) [83] postulated an important physiological role of the oxidative and non-oxidative PPP in stabilizing the redox balance system and ROS clearance in human skin cells and it also has been associated to glaucoma pathogenesis [84,85]. In accordance, transgenic *htra2^mnd2^* mice with deficient HTRA2 activity show an obvious heart enlargement with left ventricular hypertrophy accompanied by decreased mitochondrial energy supply and decreased glucose metabolism [57,58]. Therefore, HTRA2 can be assumed as important master regulator and stabilizer for the mitochondrial energy metabolism. In addition, Ding et al., (2009) [63] detected decreased amounts of mitochondria and an abnormal mitochondrial morphology in the retina of *HTRA2*-deficient mice compared to the wild-type, indicating a fundamental role of HTRA2 in the maintenance of the mitochondrial homeostasis in the eye. However, it still needs to be determined whether all these retinal proteomic changes are due to the direct interaction (inhibition) of HTRA2 with the CDR1 sequence motif *ASGYTFTNYGLSWVR* or other cell-mediated mechanism (e.g., receptor binding). The specific CDR1 peptide-protein interaction should, furthermore, be verified by more sensitive techniques, such as yeast two-hybrid systems [86] or cross-linking experiments [87]. Both methods allow the analysis of physical interactions of protein–protein complexes and, in particular, the MS-based cross-linking technology can provide important sequence information of the active binding sites of the respective proteins [87,88]. Beyond that, what needs be proven is whether the CDR-induced protein markers represent direct interaction partners of HTRA2 or if they develop as a consequence of CDR-induced downstream signaling cascades.

## 5. Conclusions

The present study shows, for the first time, that short synthetic disease-associated CDR peptides without any information about the native antigen specificity provide a great potential target for the amelioration or treatment of glaucoma. Moreover, our results support the mitochondrial serine protease HTRA2 as a key player in the pathogenesis of glaucoma, which should receive particular focus in future glaucoma research projects. This innovative procedural method could offer completely new therapeutic strategies in other immune-mediated inflammatory and neurodegenerative diseases. Beyond that, we would like to encourage other research groups to apply the established MS-based platform for CDR peptide identification [25] in combination with the present MS-based epitope identification workflow for biomarker discovery to other autoimmune-related neurodegenerative diseases in future study designs.

## Figures and Tables

**Table 1:** List of CDR-induced top canonical pathways revealed by Ingenuity Pathway Analysis (IPA).

Canonical Pathway	−Log (*p*-Value)	Molecules
Mitochondrial Dysfunction	3.37	*COX6C, ATP5MF, VDAC2*
Protein Ubiquitination Pathway	2.82	*PSMA6, HSPE1, HSP90AA1*
Oxidative Phosphorylation	2.39	*COX6C, ATP5MF*
PI3K/AKT Signaling	2.24	*YWHAE, HSP90AA1*
Thioredoxin Pathway	2.21	*TXN*
Pentose Phosphate Pathway (Non-oxidative)	2.21	*TKT*
Aldosterone Signaling in Epithelial Cells	2.03	*HSPE1, HSP90AA1*
Pentose Phosphate Pathway	2.02	*TKT*
NRF2-mediated Oxidative Stress Response	1.89	*ERP29, TXN*

**Table 2:** List of top molecular and cellular functions analyzed by Ingenuity Pathway Analysis (IPA).

Molecular and Cellular Functions	*p*-Value	Number of Molecules
Post-Translational Modification	8.59 × 10^−7^–8.59 × 10^−7^	4
Protein Folding	8.59 × 10^−7^–8.59 × 10^−7^	4
Molecular Transport	1.31 × 10^−2^–7.28 × 10^−6^	14
Protein Trafficking	1.25 × 10^−2^–7.28 × 10^−6^	6
Cellular Function and Maintenance	1.48 × 10^−2^–3.57 × 10^−5^	9

## Supplementary Materials

The following are available online at https://www.mdpi.com/2077-0383/8/8/1222/s1, Figure S1: Bar plot showing the intensity of synthetic biotin-[TTDS]-*ASGYTFTNYGLSWVR* and synthetic biotin-[TTDS]-*ASQSVSSYLAWYQQK* before and after coupling to commercially available magnetic streptavidin beads; Figure S2: Identification of potential epitope targets of the CDR1 peptide *ASQSVSSYLAWYQQK* by affinity-based proteomic strategy with 5 mg homogenized pig retina; Figure S3: Bar plots showing proteins with at least a tendency (P < 0.1) to be differentially expressed between CDR-treated retinal explants and untreated controls; File S1: Protein identifications of the affinity capture experiments; File S2: Protein identifications of the CDR-treated and untreated retinal explants; File S3: Molecular and cellular functions of significantly changed proteins (*p* < 0.05).

## Abbreviations

AAB	Autoantibodies
ABC	Ammonium bicarbonate
ACN	Acetonitrile
AGC	Automatic gain control
AMD	Age-related macular degeneration
CDR	Complementarity-determining region
CID	Collision-induced dissociation
CTRL	Control group
DE	Dynamic exclusion
DTT	Dithiothreitol
EtOH	Ethanol
FA	Formic Acid
Fab	Antigen-binding fragment
Fc	Crystallisable fragment
FDR	False discovery rate
FR	Framework region
IAA	Iodoacetamide
IOP	Intraocular pressure
IPA	Ingenuity Pathway Analysis
LC-MS	Liquid chromatography-mass spectrometry
MeOH	Methanol
MS	Mass spectrometry
ONC	Optic nerve cut
PBS	Phosphate-buffered saline
POAG	Primary open-angle glaucoma
RGC	Retinal ganglion cells
ROS	Reactive oxygen species
RPE	Retinal pigment epithelium
STRING	Search Tool for the Retrieval of Interacting Genes/Proteins
TIC	Total ion current
TFA	Trifluoroacetic acid
VH	Variable heavy chain
