# Supplementary material for: Synthetic Polyclonal-Derived CDR Peptides as an Innovative Strategy in Glaucoma Therapy
Source: J Clin Med. 2019 Aug 15;8(8):1222. doi: 10.3390/jcm8081222 (PMC6723090; doi:10.3390/jcm8081222)

**Fig. S1:** Bar plot showing the intensity of synthetic biotin-[TTDS]-*ASGYTFTNYGLSWVR* **(A)** and synthetic biotin-[TTDS]-*ASQSVSSYLAWYQQK* **(B)** before and after coupling to commercially available magnetic streptavidin beads (N=3 per group). *: Groups marked with an asterisk are presented only by one replicate.


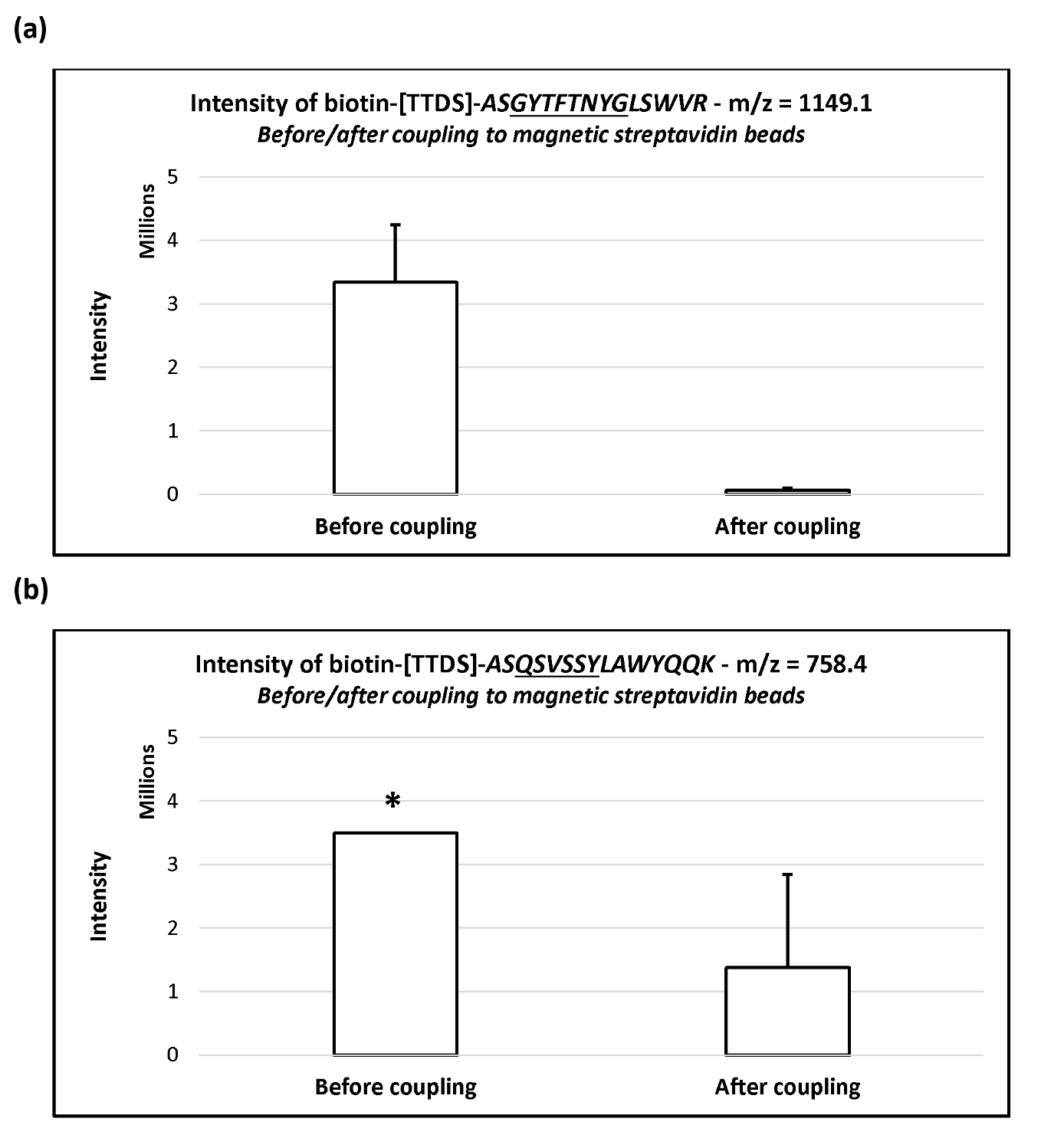


**Fig. S2:** Identification of potential epitope targets of the CDR1 peptide *ASQSVSSYLAWYQQK* by affinity-based proteomic strategy with 5 mg homogenized pig retina. Volcano plot showing log2 fold change plotted against -log10 adjusted P value for samples from CDR-labeled bead group (N=3) versus samples from control bead group (N=3) (P<0.001; log2 fold change > 3). No significant interaction partner was identified for synthetic CDR1 peptide*ASQSVSSYLAWYQQK.*


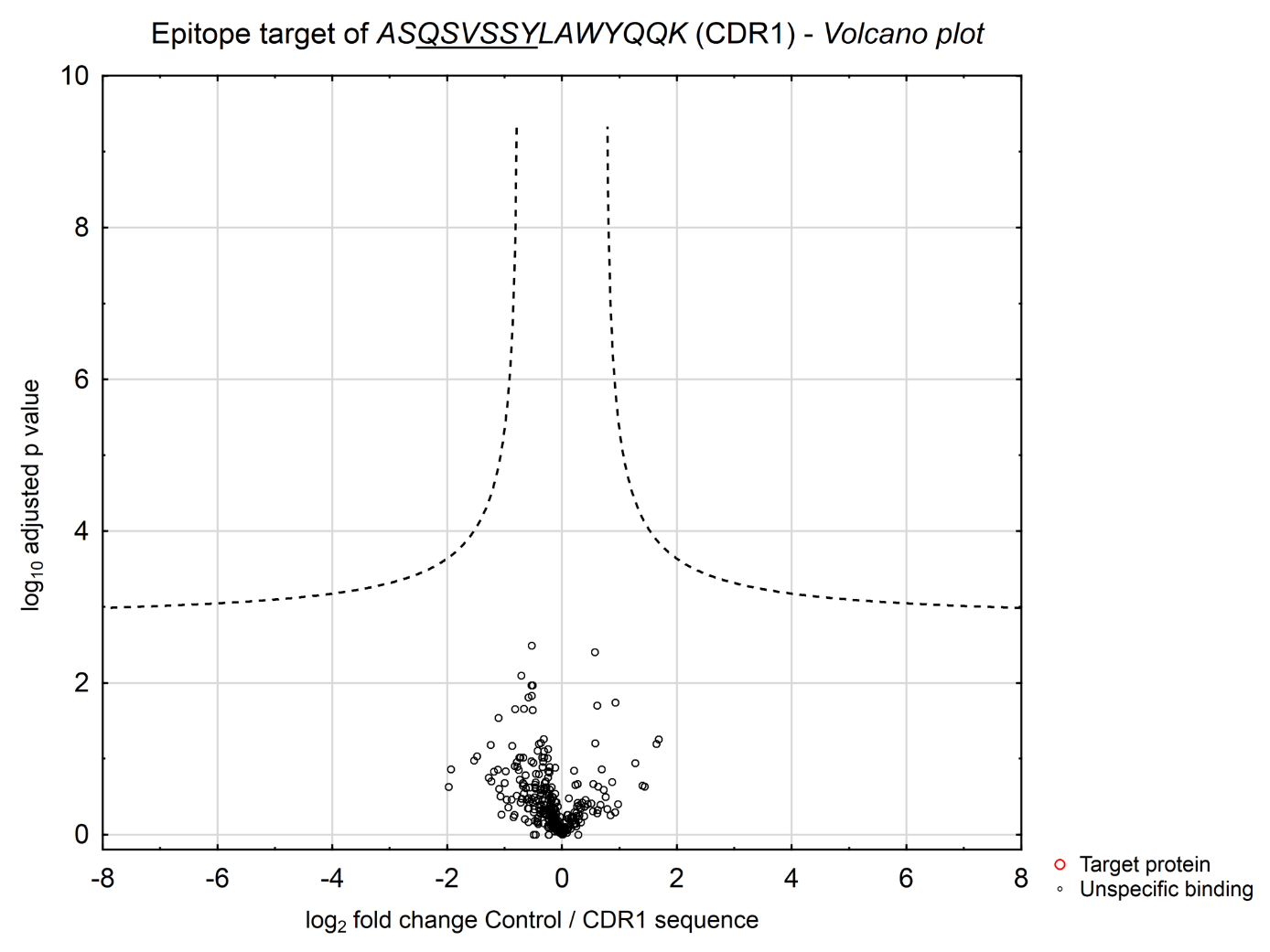


**Fig. S3:** Bar plots showing proteins with at least a tendency (P < 0.1) to be differentially expressed between CDR-treated retinal explants and untreated controls (N=3 per group). Retinal explants were cultivated either with medium without any peptide (untreated control) or with medium with 25 µg/ml CDR peptide for 24 h after optic nerve cut (ONC).


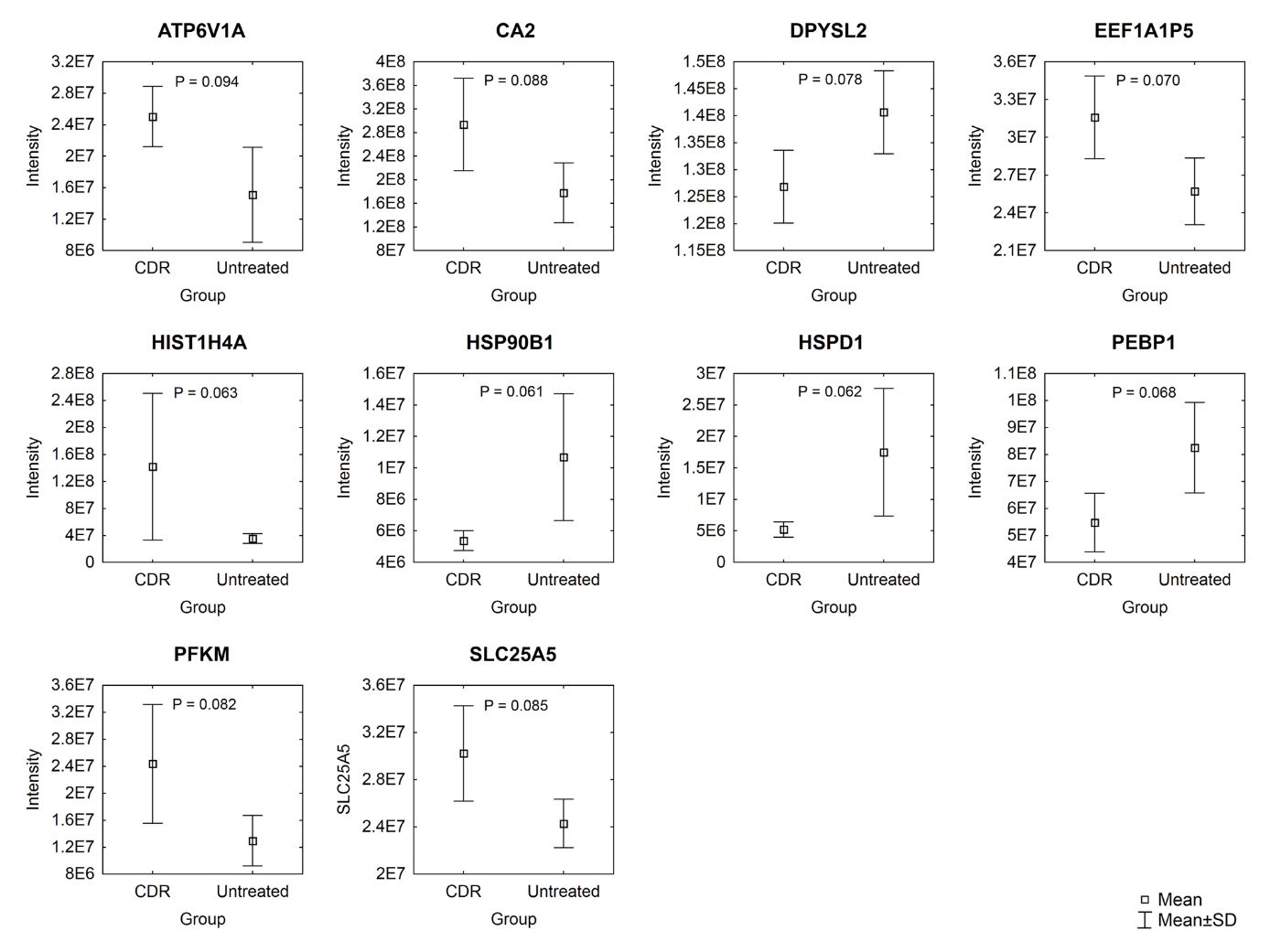

Supplement: Supplementary file 1 [file jcm-08-01222-s001.zip › supples-jcm-547972/Supplementary Figures.docx]
